# Mitigating the Goldilocks effect: the effects of different substrate models on track formation potential

**DOI:** 10.1098/rsos.140225

**Published:** 2014-11-12

**Authors:** Peter L. Falkingham, Julian Hage, Martin Bäker

**Affiliations:** 1 Structure and Motion Laboratory, Department of Comparative Biomedical Sciences, Royal Veterinary College, London, UK; 2 Institut für Werkstoffe, Technische Universität Braunschweig, Braunschweig, Germany

**Keywords:** footprint, track, finite-element analysis

## Abstract

In ichnology, the Goldilocks effect describes a scenario in which a substrate must be ‘just right’ in order for tracks to form—too soft, the animal will be unable to traverse the area, and too firm, the substrate will not deform. Any given substrate can therefore only preserve a range of tracks from those animals which exert an underfoot pressure at approximately the yield strength of the sediment. However, rarely are substrates vertically homogeneous for any great depth, varying either due to heterogeneity across sediment layers, or from mechanical behaviour such as strain hardening. Here, we explore the specificity of the Goldilocks effect in a number of virtual substrates simulated using finite-element analysis. We find that the inclusion of strain hardening into the model increases the potential range of trackmaker sizes somewhat, compared with a simple elastic–perfectly plastic model. The simulation of a vertically heterogeneous, strain hardening substrate showed a much larger range of potential trackmakers than strain hardening alone. We therefore show that the Goldilocks effect is lessened to varying degrees by the inclusion of more realistic soil parameters, though there still remains an upper and lower limit to the size of trackmaker able to traverse the area while leaving footprints.

## 2. Introduction

The formation of a track is a result of an interaction between an animal and a substrate, and as such final track morphology is determined by three factors: anatomy (of the foot), foot motion and the substrate properties [1–3]. Of these three factors, the former two determine where, how and to what extent force is applied, while the latter—substrate—governs the response to that force. This response will ultimately determine the depth of a track, which in the case of fossil tracks will affect initial preservation potential and also endurance to weathering and erosion post-exposure, influencing the probability firstly of discovery and secondly of correctly interpreting the track in terms of trackmaker and palaeoenvironment.

Experimental studies carried out with the express purpose of elucidating this relationship between substrate consistency and track morphology (particularly depth) are not a new phenomenon, dating back to the dawn of palaeoichnology in the early nineteenth century with William Buckland’s tortoise over piecrust experiments [4]. Since Buckland, numerous authors have contributed to our understanding of foot–sediment interactions. Particularly of note are those more recent studies which aimed not only to record surface morphology, but also to understand the unseen deformation beneath the surface. Plasticine [5,6], plaster of paris [7,8] and coloured cement [9] have been used to produce tracks which can be sectioned and in which subsurface deformation can be observed. Unfortunately, maintaining strict control over the mechanical properties of the substrate can be difficult in a physical experiment, and visualizing the interior of the track is a destructive process which only shows the final morphology and therefore lacks a temporal component (with the notable exception of the X-ray study described by Ellis & Gatesy [10]).

For these reasons, computer simulations have recently been introduced for use in the study of dinosaur and other vertebrate tracks. Computational work concerning track formation has predominantly employed the engineering analysis tool finite-element analysis (FEA) to simulate the interaction of foot and substrate [11–17]. Other studies have incorporated different computational techniques to look at other aspects of dinosaur ichnology such as weathering [18] or track placement according to gait [19–21]. These studies have provided insights into track formation that would be difficult or impossible to obtain through other means. In the case of the track formation studies, many of the difficulties associated with physical modelling are alleviated, that is to say track formation is dynamically visible throughout, the results can be visualized non-destructively in many ways, and substrate properties can be precisely controlled. However, the application of FEA to the study of track formation is still in its infancy, and there remains room for progress in both the indenter morphology and motions, and in increasingly more realistic substrate models.

In a recent study, Falkingham *et al*. [14] used FEA to simulate track formation over a range of substrate parameters as well as various trackmaker body masses and foot morphologies. That study illustrated what was termed the ‘Goldilocks effect’, demonstrating that in order for a track to form, a substrate must not be too soft nor too firm (given homogeneity), and importantly showed that the range of parameters in which the substrate was ‘just right’ was surprisingly small, and varied according to the trackmaker’s size and foot shape. In that study, Falkingham *et al*. [14] used the von Mises elastic–perfectly plastic model to represent a simple homogeneous mud/clay-like substrate. This model requires relatively few parameters (shear strength, *Cu*, Young’s modulus, *E*, and Poisson ratio, *v*) with which to explore variation in track formation, reducing the complexity of the problem. However, this model represents only a narrow range of naturally occurring substrates.

In this paper, we aim to explore the ways in which the ‘Goldilocks effect’ can be mitigated through additional, more complex substrate models. In particular, we incorporate plastic hardening and contact friction to the von Mises model, simulate heterogeneous substrates composed of multiple layers of differing properties, and employ the Drucker–Prager model to simulate more sand-like substrate.

## 3. Material and methods

### 3.1 Model geometry

The indenter used in this study comprised a simple cylindrical ‘foot’ measuring 125 mm in radius and 100 mm in height. The base of the cylinder was bevelled using a radius of 10 mm. The indenter was composed of linear hexahedral (8-node) and a small number of linear triangular prism (6-node) elements. We deliberately constrained our indenter morphology to be simple, rather than representative of a specific animal’s autopodia, in order to focus on the effects of the substrate properties. While previous studies have shown that foot morphology can affect track depth and morphology [13–15], these effects are relatively subtle compared with the changes in track depth resulting from changing substrate properties. The size was representative of a small to medium sauropod.

**Figure 1.**
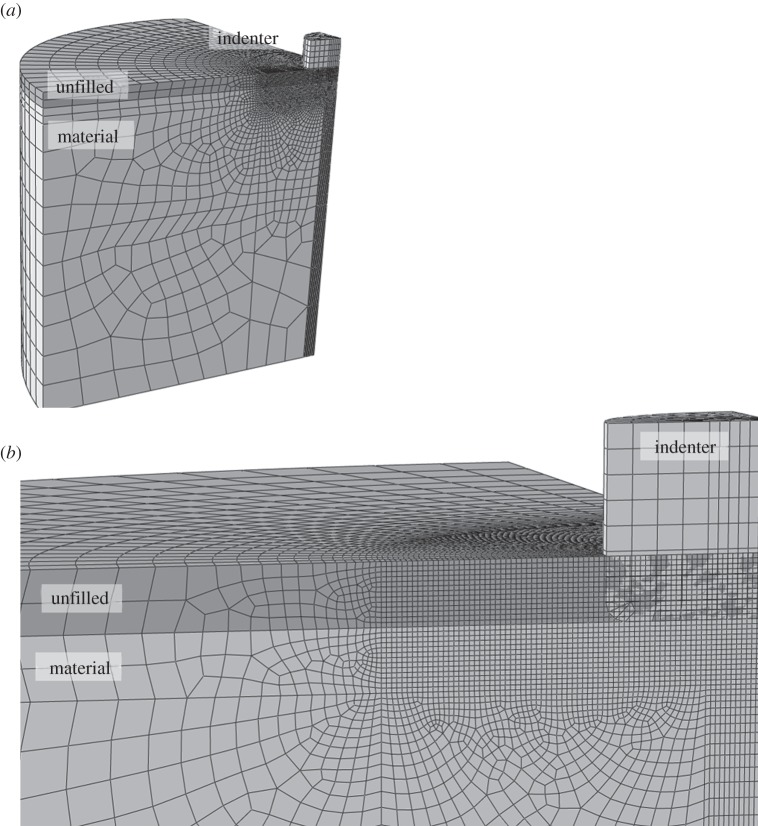
The mesh used to model the substrate and indenter. The radius of the virtual substrate was 1.1 m, and the indenter was 125 mm in radius (bevelled 10 mm at the base).

In principle, the indenter model could be axisymmetric but was modelled in three-dimensions here to allow generalization to more realistic foot shapes in later projects. However, the symmetrical nature of the model was used to save computer time by only calculating one quarter of the model. The substrate comprised a cylindrical volume measuring 1.1 m in height and radius (of which a quarter was simulated). The large radius and depth of the substrate compared with that of the indenter was used in order to avoid significant boundary effects. The substrate mesh was fixed in all degrees of freedom at the base and external lateral side, and symmetry boundary conditions were used at the two planes of symmetry (figure 1). In order to maintain resolution but reduce computation time, a finer mesh was used in the area where the indenter would contact the substrate, while elements became progressively larger at depth, as in Falkingham *et al.* [11] and subsequent studies (figure 1).

Unlike the previous studies by Falkingham *et al*. [11–15] in which the elastic–perfectly plastic nature meant that failure to converge computationally was assumed to equate with substrate failure, the incorporation here of strain hardening and friction (see below) meant that the indenter could reach considerable depths without complete substrate failure. Standard finite-element (FE) techniques will suffer from mesh distortions, and thus convergence problems, when large deformations are involved. To avoid this, an Eulerian technique was used (Dassault Systems (2011), ABAQUS 6.11 Analysis Users Manual) in which the mesh is spatially fixed and material flows through the mesh. A comparison with a standard Lagrangian technique showed no significant deviations for indentation depths of 10 mm or less, and previous work has demonstrated the feasibility of the Eulerian approach to such models [22,23]. Because the mesh is stationary and material flows through the mesh in an Eulerian simulation, it is necessary to create a mesh also in those regions that may be filled by the material as it is deformed. This region is marked as ‘unfilled’ in all plots (figure 1). In the initial state, the indenter overlaps with the unfilled region but not with the material. The substrate was composed of 8-node linear Eulerian hexahedral elements.

Since the simulation was carried out using an explicit FE model (due to the use of Eulerian elements in ABAQUS), elastic waves may occur in the foot on initial acceleration. To avoid this, all nodes of the foot were simultaneously accelerated instantly to a speed of 0.5 m s^−1^ in a first step before the foot contacts the substrate. At the beginning of the second step, this boundary condition was released on all nodes except those at the top of the foot. A comparison with an implicit static model was performed for small indentation depths to ensure that the influence of elastic waves on the indentation force is small. True contact using Coulombian friction was assumed between the foot and the ground so that relative movement between the foot and the ground was possible. Different values of the friction coefficient were studied (see below).

### 3.2 Material properties

The indenter was composed of an elastic material with Young’s modulus of 5 GPa and Poisson’s ratio of 0.4. The very high Young’s modulus of the indenter relative to the substrate (see below) made the indenter essentially undeformable. Six experiments were carried out in which the substrate and the coefficient of friction were systematically altered. The properties and substrate models used in these experiments are summarized below.

The substrate was assumed to be elasto-plastic, with a Young’s modulus of 50 MPa and a Poisson’s ratio of 0.4. For the plasticity of the ground, different models were used.

**Figure 2.**
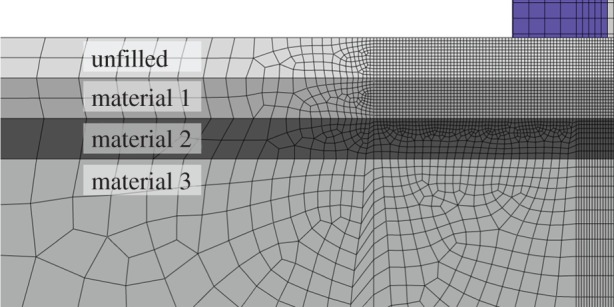
The distribution of material properties for experimental set-up 4 (heterogeneous substrate). Materials 1 and 2 are 5 cm thick, material 3 extends to the base of the substrate.

1. Ideally plastic von Mises plasticity with yield strength of 50 kPa, incorporating (i) no friction, (ii) a friction coefficient of 0.2 and (iii) a friction coefficient of 1, between the indenter and the substrate.
2. Strain hardening von Mises plasticity with 50 kPa yield stress and 70 kPa flow stress at 100% plastic deformation. Indenter–substrate friction coefficient was 0.2.
3. A Drucker–Prager model with material angle of friction 15^°^, flow stress ratio *K*=1, dilation angle 0, and shear hardening with 50 kPa yield stress and 70 kPa flow stress at 100% plastic deformation. Indenter–substrate friction coefficient was 0.2.
4. A three-layered model, where all layers were plastically hardening, but with the top layers being softer than the lower layers. The three materials were assumed to be plastically hardening with von Mises plasticity and the following properties:
  material 1: 50 kPa yield stress and 70 kPa flow stress at 100% plastic deformation.
  material 2: 70 kPa yield stress and 100 kPa flow stress at 100% plastic deformation.
  material 3: 100 kPa yield stress and 140 kPa flow stress at 100% plastic deformation.
  The thickness of the top two layers was 5 cm each (figure 2), and the friction coefficient between the indenter and the substrate was 0.2.

The first model is identical to that used in previous studies by Falkingham *et al.* [11,13–15] and represents a simple, idealized mud which behaves homogeneously. The friction used in this model refers to the friction between indenter and substrate, and was not a part of the soil model itself.

Model 2 incorporated strain hardening into model 1—that is, as the substrate was loaded, it became progressively harder to deform. Strain hardening is well documented in the soil mechanics literature [24–26] and was recently incorporated into footprint simulations [16].

The Drucker–Prager model is similar to the Mohr Columb model used by Schanz *et al*. [16], though differs in representing a smooth cone in three-dimensional principal stress space, rather than a hexagonal cone. Because a friction parameter is incorporated, it is often used in soil mechanics to simulate sands.

Finally, model 4 represents a homogeneous sediment volume composed of three layers. Each layer uses model 2, albeit with increasing yield stress at deeper levels. This represents a substrate in which deeper layers have become compressed and accordingly more resistant to deformation.

In all models, the initial yield stress is 50 kPa, which represents a ‘firm’ clay [14,25]. Young’s modulus was set to 1000 times this value [14,27]. The electronic supplementary material, figure S1, provides an indication as to the effects on the load–displacement curve of increasing or decreasing yield strength, Young’s modulus and friction angle.

## 4. Results

**Figure 3.**
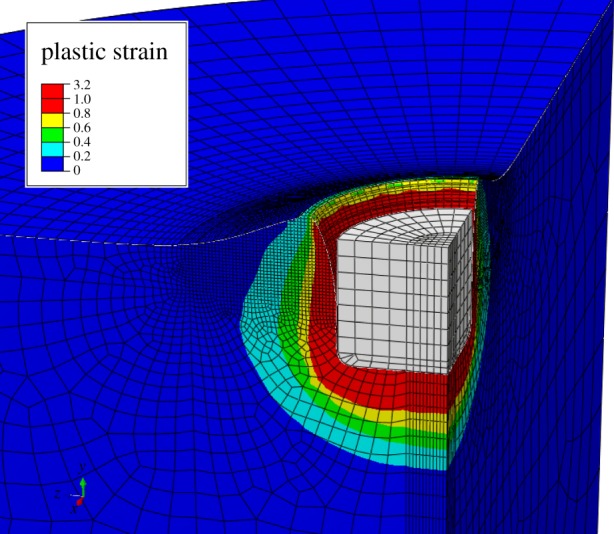
The resultant deformed substrate after indentation of the ‘foot’ to a depth of 1 radius (125 mm). The ‘unfilled’ portion of the substrate mesh is not visible, in order to show the deformation at the surface of the substrate.

Figure 3 shows the plastic strain for the ideally plastic case with no friction after the indenter has moved by a distance of one foot radius. Owing to the Eulerian technique used, the simulation was successful with no convergence problems due to mesh distortions (note that the ‘unfilled’ portion of the mesh is not shown). The Eulerian technique used enabled displacements far greater than those achieved in previous FEA track studies.

**Figure 4.**
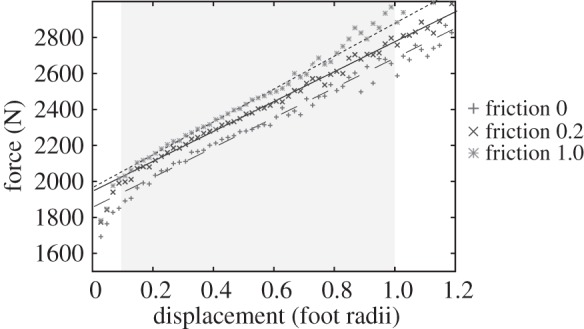
Force–displacement curves for simulation 1a, 1b and 1c (ideal von Mises elastic–plastic with foot-sediment contact friction coefficients of 0, 0.2 and 1.0). The displacement is measured in units of the foot radius, i.e. a displacement of 1 corresponds to an indentation depth of 0.125 m. Also shown are linear fits to the curves, fitted using the region between a displacement of 0.1 and 0.8 where the force–displacement curve is approximately linear.

By plotting the force–displacement curves, we are able to visualize the effect substrate parameters have on the range of forces required to produce tracks. A steeper slope covers a wider range of forces over the same indentation depths than does a shallower slope, while a higher curve indicates more force is required for a track of a given depth than a lower curve. At larger displacements, some oscillations in the force are visible (figure 4). These are due to the coarser mesh at depth—the Euler algorithm causes a force maximum when a new element row is reached. Furthermore, at large deformations, some material leaves the simulation region (the mesh part marked ‘unfilled’ in figures 1 and 2), leading to a reduction in the deformation force.

### 4.1 Varying coefficient of friction

Figure 4 shows the force–displacement curves for the three values of friction coefficient used in the ideally plastic material (simulations 1a, 1b and 1c). The force increases approximately linearly over a wide range because the amount of material being plastically deformed increases with indentation depth. As can be seen from the data, altering the friction has little effect on the slope, but an increased friction coefficient does require greater overall force in order to indent the foot to the same depth (i.e. to indent the foot to 62.5 mm depth, 1/2 radius, a frictionless contact requires approx. 2280 N, but a friction coefficient of 1 requires approx. 2410 N).

### 4.2 Varying substrate model

**Figure 5.**
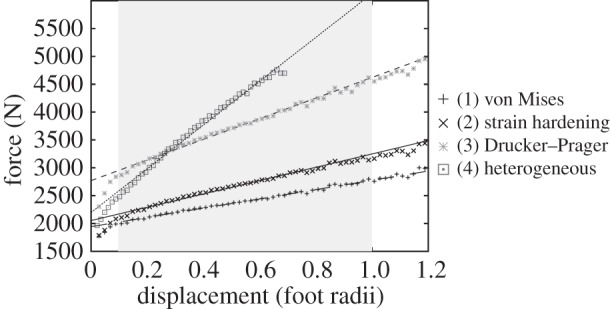
Force–displacement curves for simulations 1b and 2–4; ideal plastic, strain hardening, Drucker–Prager and heterogeneous layers. The friction coefficient was assumed to be 0.2 in all cases. Also shown are linear fits to the curves, fitted using the region between a displacement of 0.1 and 0.8, where the force–displacement curve is approximately linear.

The force–displacement curves for each substrate model (i.e. simulations 1–4, coefficient of friction = 0.2) are shown in figure 5. Linear fits were performed for the same displacement range, except for the case of heterogeneous soil model where large oscillations in the force occurred at a displacement of 0.7 foot radii.

As can be seen in figure 5, the slope of the linear fit increases when material hardening is taken into account. The inclusion of strain hardening increases the slope slightly in relation to the ideal elastic–plastic condition. The Drucker–Prager model increases both the slope and the required force needed to deform the substrate, while the heterogeneous model in which three materials were layered shows a considerably steeper slope. Initially, the heterogeneous model requires less force than the Drucker–Prager model in order to indent the substrate, but as the depth to which the foot sinks becomes greater, subsequently stiffer materials are reached and greater forces are required.

**Figure 6.**
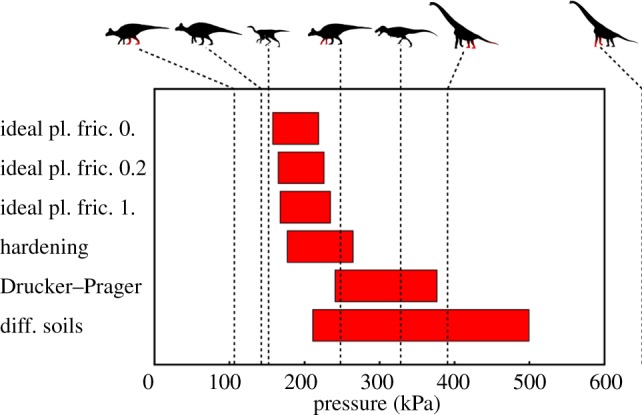
The possible ranges in underfoot pressure that could produce tracks between 0.1 and 1 foot radius in depth, based on the linear fit from figure 5. In the case of the ideal elastic–plastic model, this range is very constricted, as in Falkingham *et al*. [14], regardless of how the foot–sediment frictional contact was modelled. The potential pressure ranges increase somewhat for our strain-hardening and Drucker–Prager simulations, and increase considerably for the heterogeneous model.

The linear fits indicate the forces required to indent the substrate to a specified depth. If a range of possible depths is specified, the range of forces that can produce such tracks can be read from the graphs. For the purposes of this paper, we considered 0.1 foot radii as a minimum value, as lower values would be extremely shallow (for our 125 mm radius foot, this depth would be only 12.5 mm) and would therefore be highly susceptible to discovery bias or weathering and erosion [18]. We used 1 foot radius as a maximum depth, as very few fossil tracks are reported sinking to depths greater than half the foot width—in the cases where this does occur (e.g. [28]), tracks can tend to seal up or collapse. Using these bounds in conjunction with the linear fits to our simulations, we plotted the possible ranges of applied pressure that could leave tracks on the equivalent substrates (figure 6). Not all workers may agree that 1 foot radius is a suitable maximum depth as this is a subjective limit based on the experience of the authors, and as such we note that readers wishing to consider the results of this manuscript in context of deeper tracks may use the linear fits of figures 4 and 5 to extrapolate to any other maximum relative depth. However, note that for very large indentation depths of several radii, the linear fit will become increasingly imprecise.

Because the indenter has a known surface area in contact with the substrate, we can calculate the range of pressures required to produce tracks in the range of 0.1–1 foot radius in depth. Given that force can be seen to increase linearly with indentation depth, the force–yield strength relationship can be described by the following equation:

$$F=F_{Y}+m\mu ,$$

where *F* is the force applied, *F*_*Y*_ is the yield force, *m* is the slope of the graph and *μ* is the depth of indentation. Dividing this by foot area converts this to an equation for pressure

$$P=Cu+\left(\frac{4}{\pi }\right)\;\left(\frac{m}{r}\right)\;\left(\frac{\mu }{r}\right),$$

where *P* is the pressure applied, *Cu* is the yield strength, *r* is the radius of the foot and *μ*/*r* is now the dimensionless indentation. Applying this to the boundaries of minimum and maximum indentation depth noted above (i.e. *μ*/*r*=0.1, 1), we calculated the minimum and maximum pressures required to produce a visible footprint, independent of the size of the hypothetical trackmaker (figure 6).

## 5. Discussion

Our results indicate that by increasing the complexity and realism of a soil model in an FE analysis, the ‘Goldilocks effect’ can be mitigated, in some cases to a great extent. The pressure ranges required to produce visible tracks, while not exceeding a value above which a trackmaker would become mired (or avoid the area), are considerably larger in those simulations in which strain hardening, the Drucker–Prager model, or heterogeneity are incorporated.

In their original study, Falkingham *et al.* [14] illustrated the effect with an ideal-plastic von-Mises FE model, and this resulted in extremely narrow ranges of potential pressures for any given soil; however, those authors speculated on the effects of multi-layered sediments, or sediments in which yield strength increased with depth, noting ‘…that this being the case, there is a much larger range of possible track-bearing substrates…’ [14], p. 1148. In this regard, our study here does not contradict the previous work, but rather expands upon it, presenting more real-word applicable results. The ‘Goldilocks effect’ is therefore still an applicable term, and ranges of required pressure for track formation are still limited at both the small and large extremes for any given substrate. The extent of that range is highly dependent upon substrate properties.

The simulations in which the coefficient of friction was altered showed only a small effect, increasing the required force to produce tracks only slightly. Because the slope of the force–displacement curves remained essentially unchanged, the Goldilocks effect remained constant in terms of the range of forces required to produce tracks. Strain hardening had a more noticeable effect on the force–displacement curve, increasing the slope such that the range of forces required to produce tracks of comparable depth to those in a non-hardening substrate was larger. This directly translates to such a substrate being able to record a more diverse range of animal sizes.

The model in which multiple layers were simulated, each with progressively stiffer, strain-hardening soils, had the greatest range of different foot pressures able to create visible footprints, in agreement with the expectations stated in Falkingham *et al*. [14]. Even with this much larger range of potential trackmakers than in Falkingham *et al*. [14], the potential diversity recorded in tracks formed in such a substrate is limited. Using the taxa and associated foot pressures from the previous study, only the *Giraffatitan*, *Tyrannosaurus* and *Edmontosaurus* would leave impressions between 0.1 and 1 foot radius in depth. Of these taxa, the quadrupedal *Edmontosaurus* would only leave manus impressions—the pressure produced by the pedes being too little to impress into the substrate. The *Giraffatitan*, conversely, would be unable to traverse such a substrate as the manus would sink too deeply due to the considerably higher underfoot pressures. As such, even this substrate with the widest range of potential trackmakers would, in reality, only record the *Tyrannosaurus* and a manus-only *Edmontosaurus* track from the four taxa. If different soil properties are assumed, the absolute value of the pressure range that will produce footprints will change, but the relative size of the range will not. To increase the range so that trackmakers with widely differing sizes can simultaneously produce tracks, an even stronger gradient of the material properties will probably be required so that small trackmakers indent the softer top layer and large trackmakers sink through to the harder lower layers.

However, these animals are morphologically very different, and span a range of body masses from 400 to 26 000 kg. Instead, if we consider a single form that scales isometrically, we can explore the range of sizes possible for a given taxa because pressure is directly proportional to length. As an example, consider a bipedal animal of length *L* that produces an underfoot pressure of 211 kPa, directly at the lower end of the allowed pressure range for the model with different soils. An isometrically scaled animal of 2.4 times its size would then be able to produce a footprint with a depth of one foot radius. The indentation depth of the larger animal would then be 24 times larger than that of the smaller one. If our smaller animal in this case were a 5 m theropod, then this substrate would support animals up to 12 m in length. However, the tracks of the smaller theropod would be around 0.011 m in depth, while those of the larger animal would be 0.27 m deep (note that this is deeper than any reported theropod track of this size, indicating our 1 foot radius limit may be generous). This scenario clearly caters for the intuitive idea that larger, heavier animals leave deeper tracks, and is in line with recent attempts to back-calculate animal mass from the depth of fossil tracks [16].

## 6. Conclusion

This study has expanded upon the earlier study by Falkingham *et al.* [14], incorporating additional complex, more realistic soil models to explore what was previously termed ‘the Goldilocks effect’ in which only a narrow range of animals are able to leave tracks in a given substrate. Our study has shown that incorporating realistic effects such as strain hardening, or vertical heterogeneity, or by using different soil models, the narrow range can be considerably expanded. Even so, in cases where the sediment is deep enough, there will still be a range of potential underfoot pressures, and therefore animal sizes, that can deform a substrate enough to form a track but not so much as to become mired.

However, this theoretical framework still excludes extraneous confounding factors such as time averaging, lateral sediment heterogeneity and post-formational factors including fossilization, weathering, erosion and likelihood of discovery. On top of these abiotic factors affecting potential tracksite diversity, limb dynamics (motions and forces) are known to play an important role in determining the morphology—including depth—of tracks [17,28–31].

In order to expand upon these findings further, future work should incorporate yet more realistic soil models, based on specific real-world substrates (e.g. [16]). More importantly, simulations should incorporate the dynamics of the foot, and in this regard may be linked with current research into biologically and mechanically accurate FEA models of feet [32].

## Supplementary Material

Figure S1: Diagramatic representation of the effect of increasing and decreasing the Yield strength, Youngs modulus, and Friction angle on load-displacement curves
