# Supplementary figures and images for: Mitigating the Goldilocks effect: the effects of different substrate models on track formation potential
Source: R Soc Open Sci. 2014 Nov 12;1(3):140225. doi: 10.1098/rsos.140225 (PMC4448835; doi:10.1098/rsos.140225)

Red = increase variable  
blue = decrease variable

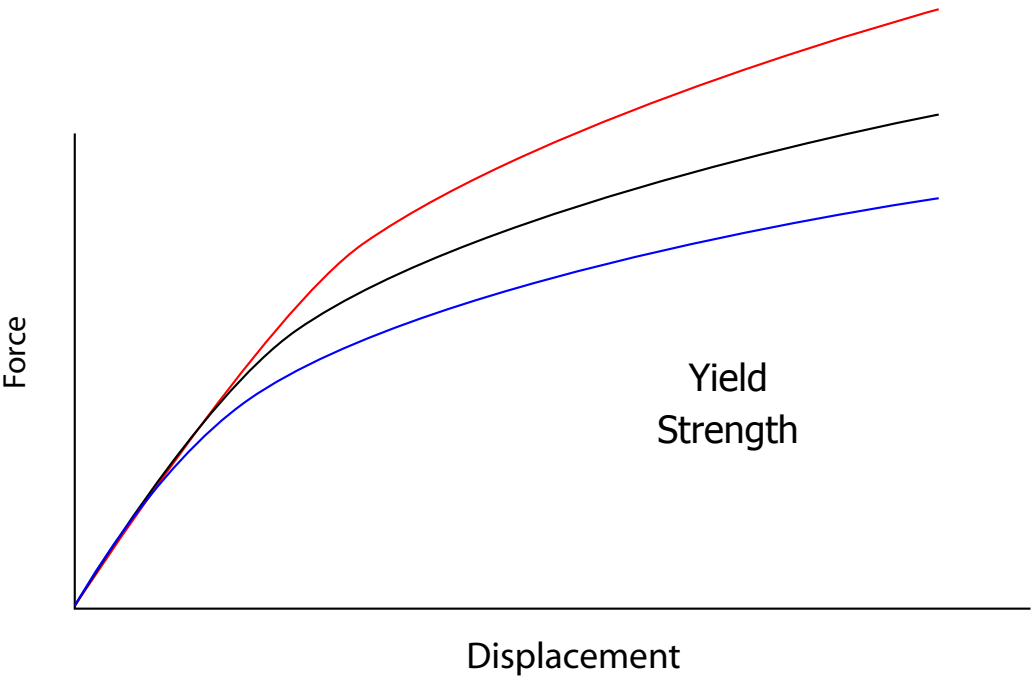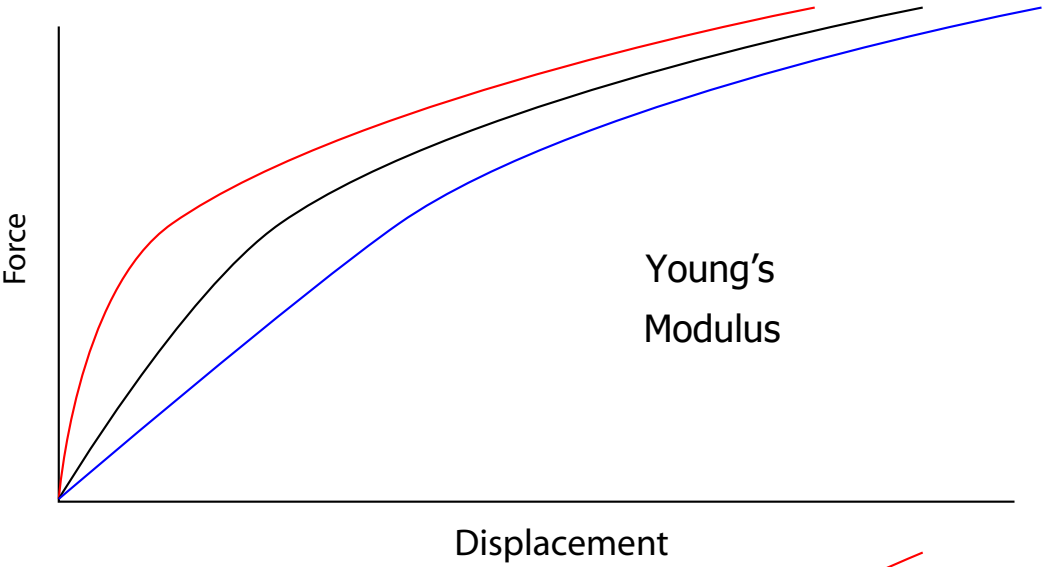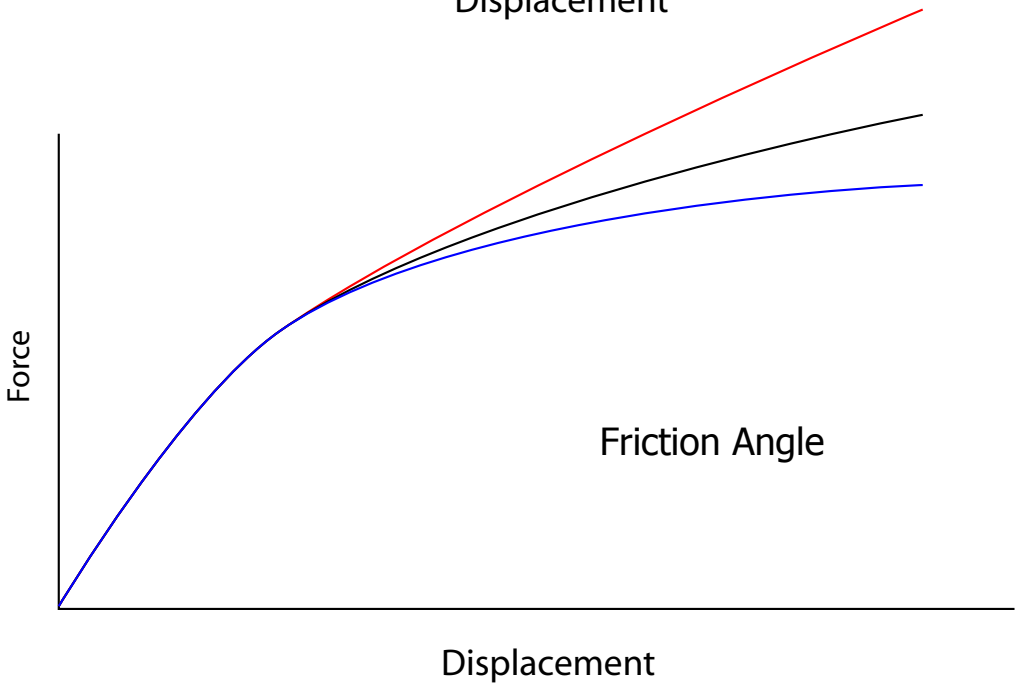

Supplement: Figure S1: Diagramatic representation of the effect of increasing and decreasing the Yield strength, Youngs modulus, and Friction angle on load-displacement curves [file rsos140225supp1.pdf]
